# Identifying training modalities to improve multitasking in older adults

**DOI:** 10.1007/s11357-014-9688-2

**Published:** 2014-07-30

**Authors:** Bianca Bier, Chloé de Boysson, Sylvie Belleville

**Affiliations:** 1 Research Centre, Institut Universitaire de Gériatrie de Montréal, 4565 Chemin Queen-Mary, Montreal, Québec H3W 1W5 Canada; 2 Department of Psychology, University of Montreal, Montreal, Quebec Canada

**Keywords:** Attentional training, Divided attention, Multitasking, Aging

## Abstract

Studies that have measured the effects of attentional training have relied on a range of training formats, which may vary in their efficacy. In particular, it is unclear whether programs that practice dual-tasking are more effective in improving divided attention than programs focusing on flexible allocation priority training. The aims of this study were as follows: (1) to compare the efficacy of different types of attentional training formats and (2) to assess transfer to distal measures. Forty-two healthy older adults were randomly assigned to one of three training groups. In the SINGLE training condition, participants practiced a visual detection and an alphanumeric equation task in isolation. In the FIXED training condition, participants practiced both tasks simultaneously with equal attention allocated to each. In the VARIABLE training condition, participants varied the attentional priority allocated to each task. After training, all participants improved their performance on the alphanumeric equation task when performed individually, including those in the SINGLE training condition. Participants in the FIXED training condition improved their divided attention, but only the participants in the VARIABLE training condition showed a greater capacity to vary their attentional priorities according to the instructions. Regarding transfer, all groups improved their performance on the 2-back condition, but only the VARIABLE and FIXED conditions resulted in better performance on the 1-back condition. Overall, the study supports the notion that attentional control capacities in older adults are plastic and can be improved with appropriate training and that the type of training determines its impact on divided attention.

## Introduction

Because we live in complex environments, divided attention is constantly required in our everyday lives. Having a conversation with the passenger while driving a car, planning and executing responses to avoid a collision, or crossing the street while talking on a hands-free cell phone are a few examples of daily activities that require divided attention between two or more concurrent tasks. There is abundant evidence indicating that older adults have more difficulty in performing two tasks concurrently (Anderson et al. 2000; Hartley and Little 1999; McDowd and Shaw 2000; Salthouse et al. 1984; Verhaeghen 2011; Verhaeghen and Cerella 2002; Verhaeghen et al. 2003). The age-related decline in divided attention and attentional control has been associated with several negative outcomes later in life. These outcomes include falling (Faulkner et al. 2007; Gaspar et al. 2013) and automobile collisions (Daigneault et al. 2002). Finding ways to improve divided attention abilities could therefore have a significant impact on the daily living activities of older adults. However, training programs may differ in their ability to improve attentional control in healthy older adults and to promote transfer to untrained tasks. The present paper pursues two broad objectives: (1) comparing three different attentional training formats to select the most efficient training modalities and (2) assessing transfer to distal and proximal measures to identify training strategies that lead to meaningful cognitive improvements.

Divided attention is part of the attentional control capacities. Attentional control (Baddeley and Hitch 1975; Norman and Shallice 1980) refers to the ability to coordinate and monitor information processing and relies on a set of distinct cognitive processes including inhibition, task switching, and dividing and modulating attention (Baddeley 1996; Miyake et al. 2000). These processes allow one to select the most efficient strategy with which to complete a task based on environmental demands. Among the different attentional control capacities, divided attention represents a potentially critical target for cognitive training. First and as mentioned above, its impairment can have an impact on different dimensions of everyday life. Second, this is an area of frequent complaints among healthy older adults (Langlois and Belleville 2013; Weaver Cargin et al. 2007). Indeed, one of the most frequent complaints is a decreased capacity to memorize or learn new things while in an attention-demanding environment (Langlois and Belleville 2013).

There is increasing evidence that carefully designed training strategies can lead to meaningful improvements in attention. It is however unclear which components optimize the therapeutic effects in older adults because of the large number of training programs that have been used. Studies have aimed to train divided attention in older adults and examine how training the ability to modulate attention according to task demands differs from training a static division of attention. These training protocols are known as variable-priority training (VP) and fixed-priority training (FP), respectively. Specifically, FP training consists of performing the two tasks simultaneously while allocating the same amount of attention to each task; VP training requires participants to modulate their attentional priority by emphasizing performance on one task over the other. The level of attention allocated to each task varies throughout the training.

It has been proposed that VP training may be more effective than FP training in improving dual-task coordination and enhanced attentional control, because participants are trained to manage competing task priorities through self-regulation of their attentional priorities. Indeed, studies have reported enhanced dual-task coordination and attentional control following VP training compared to FP training (Gagnon and Belleville 2012; Kramer et al. 1995; Lee et al. 2012; Voss et al. 2012). For instance, Kramer et al. (1995) evaluated the effects of three 1-h sessions of FP and VP training using a visual monitoring task and an alphabet-arithmetic task. They found that both groups improved their ability to divide attention but that the gain was greater for those that received VP training. Gagnon and Belleville (2012) compared the effects of six 1-h sessions of VP and FP training in people with mild cognitive impairment. Importantly, the authors added a self-regulatory strategy to the VP training condition in order to favor metacognition, which has been suggested to be critical for intervention success (Clare et al. 2004). They found that after training, only the VP training group had a reduction in performance costs associated with dual-task performance, suggesting a unique benefit of VP training. Lee et al. (2012) and Voss et al. (2012) used a complex video game (Space Fortress, Donchin 1995) to compare the efficacy of FP and VP training in young adults. Participants in the VP training group were asked to modulate their attention to different components while playing the game (e.g., control the movement of their ships, monitor the number of times they shot the enemy, and monitor their ability to gain a bonus). Participants in the FP training group were asked to maximize performance and focus on obtaining the highest total score by emphasizing each task component equally. In both studies, better game mastery and skill acquisition were found in the VP training group.

In contradistinction, Bherer et al. (2005, 2008) failed to obtain superiority for VP training over FP training. They assessed VP and FP training using simultaneous visual (letter) and auditory (tone) discrimination tasks. The training groups were compared to a no-contact control group, which performed only the pre- and post-training sessions. The authors found improvement in divided attention abilities for both training groups, but not for the no-contact control group. Importantly, there was no additional benefit for the VP group compared to the FP group. One possible reason why Bherer et al. (2005, 2008) failed to replicate the benefits of VP training over FP training could be that the two tasks were relatively simple. The use of these simple discrimination and computer-paced tasks may have reduced the coordination requirement of the task, hence downplaying the relevance for attentional control training. Studies showing superior effects of VP over FP training used relatively complex tasks that were self-paced. These tasks might be more amenable to variations in attentional control.

Another point of difference is that many of these studies have used a 50-50 dual-task emphasis condition (i.e., allocating the same amount of attention to each task) as their critical outcome variable (Bherer et al. 2005, 2008; Gagnon and Belleville 2012; Kramer et al. 1995). This condition was used in these aforementioned studies, as their objective was to assess the effect of different training formats on dual-tasking. However, our goal in the present study was different, as our main aim was to measure the effect of different training modalities on controlled attention and modulation capacities, with an analysis of attentional priority instructions. Complying with priority instructions was considered as an instance of real-life conditions in which individuals are required to vary their attentional priority in response to environmental demands. Our paradigm differentiates the effect of training on divided attention abilities (or dual-tasking) from the effect of training controlled attention abilities. This was considered crucial, as aging has been associated with increased difficulty in the ability to flexibly allocate attentional resources. Be this as it may, our paradigm will include a condition that will require equivalent emphasis on both and this will allow measuring dual-tasking per se.

Another important and disregarded issue is whether improving efficiency on each of the single constituent tasks improves the ability to combine them. Theories of attentional control indeed postulate that combining tasks that are automatized is not as demanding as combining tasks that are new (Shallice 1994). It is therefore possible that some of the improvement in dual-task performance was due to an increased ability to perform each task in isolation. Accordingly, developing expertise by practicing two separate tasks in isolation (full attention) should result in improvements when performing the tasks simultaneously. The specific impact of training two tasks in isolation, and the way this affects performance when the two tasks are combined, remains poorly understood. Indeed, very few studies (Bherer et al. 2005, 2008; Gagnon and Belleville 2012; Kramer et al. 1995) have included a full attention training condition.

Another important issue is whether the effects of training transfer to performance on untrained tasks. According to the taxonomy proposed by Barnett and Ceci (2002), transfer can be qualified as *near* or *far. Near transfer* involves transfer to tasks that share a similar context, whereas *far transfer* involves transfer to tasks that are dissimilar. The extent to which dual-task training benefits the performance on untrained tasks is not clear. Although some studies have reported significant near transfer (Bherer et al. 2005, 2008; Boot et al. 2008; Kramer et al. 1995; Lee et al. 2012; Lussier et al. 2012) and far transfer effects (Bherer et al. 2005, 2008; Gagnon and Belleville 2012) following divided attention training, others have reported no convincing evidence of transfer effects following working memory or complex task training (Dahlin et al. 2008; Green and Bavelier 2008; Owen et al. 2010).

As discussed by Lövdén et al. (2010), the assessment of transfer effects is challenging, as the selection of both the transfer tasks as well as the appropriate control group comparisons necessitates a precise understanding of the mechanisms of action of training programs, most of which are still largely unknown. A detailed analysis of the cognitive components and strategies involved in both the training programs and the transfer tasks is indeed necessary in order to predict transfer effects and thus cognitive plasticity.

Here, one might expect that the training program involving larger metacognitive abilities (e.g., VP training) would result in a larger transfer effect than the training program that only relies on repeated practice. Indeed, some studies have reported larger near transfer effects (Kramer et al. 1995; MacKay-Brandt 2011) for VP training compared to FP training. At the same time, both Bherer et al. (2005, 2008) and Gagnon and Belleville (2012) found similar transfer effects for both VP and FP training. In turn, some studies have failed to observe far transfer effects from either type of training (Lee et al. 2012; MacKay-Brandt 2011). Thus, whether VP and FP training differ in terms of their ability to transfer from untrained tasks remains to be elucidated.

In summary, previous studies have shown that attentional training programs can improve attentional capacities in older adults; however, conditions that are most favorable remain to be better understood. First, it is unclear whether this improvement is more effective with FP or VP training. Second, it is not clear whether simultaneous dual-task training results in improvement over and above that reached by practicing each constituent task. Another important question is how different training modalities impact transfer effects to untrained tasks; specifically, whether VP, FP, or single-task training promotes near- or far-transfer effects.

To address these questions, we randomly assigned participants to one of three training groups in which they learned to perform a simple visual detection task and a complex alphanumeric equation task. The first group trained on the two tasks separately (SINGLE). The second group trained on both tasks simultaneously and was told to allocate equal amounts of attention to both tasks (FIXED). The third group trained on both tasks simultaneously but was instructed during training to modulate the amount of attention given to each task on a trial-by-trial basis (VARIABLE). In order to create an experimental design akin to real-life conditions in which individuals need to flexibly allocate their attention across tasks, we administered a combination of tasks that vary in their level of complexity and attentional demand. In daily life, it is indeed frequent that one has to divide their attention between tasks that differ in terms of how complex and “attractive” they are. For example, this is the case when a skilled driver engages in a complex conversation.

Efficacy was measured with a near-transfer task where the same task was used as in training, but with different stimuli. We expected that performance on both tasks in isolation would improve in all three groups after training. More importantly, we expected that the FIXED training group would improve their ability to divide attention by lowering their overall dual-task cost on both tasks after training, regardless of emphasis instructions. As for the VARIABLE training group, we anticipated that participants would improve their controlled attention abilities (i.e., changing attentional priorities in response to specific environmental demands). Thus, the performance of the VARIABLE training group should differ as a function of attentional allocation priority instructions after training. As a result, we expected a lower dual-task cost on the alphanumeric equation task when this task was asked to be emphasized (80 % Equation) and lower dual-task cost on detection when this task was asked to be emphasized.

To measure far-transfer effects, we used a working memory task (N-back task with a 1-back and 2-back condition). The N-back task was selected to measure transfer, as it is thought to require attentional control, particularly the ability to flexibly update working memory content and to manage proactive interference, an instance of coordination and monitoring capacity (McCabe and Hartman 2008; Miyake et al. 2000). N-back requires interleaving different subtasks: processing incoming information, maintaining activation of recently processed and potentially relevant information, and discarding recently processed but irrelevant information. Our hypothesis was that VARIABLE training and FIXED training, to a lesser extent, improved these abilities and would transfer to performance on the N-back task. We hypothesized a larger transfer to the 2-back than to the 1-back condition given that it is more demanding at the executive level.

## Method

### Participants

Forty-two healthy older adults participated in this study. All participants were recruited in the community through advertisements in retirement centers and magazines for seniors. They underwent a telephone interview to provide initial selection information. Participants were included if they were French-speaking and community-dwelling, living in the Montreal area, right-handed, and had normal or corrected-to-normal hearing and vision. Exclusion criteria included the following: alcoholism or substance abuse, presence or history of a neurological disorder or stroke, presence or history of a severe psychiatric disorder (e.g., depression, schizophrenia, and bipolar disorder), and general anesthesia in the past 6 months. Eligible persons were invited to come to the laboratory for a standardized clinical and neuropsychological battery in order to evaluate their clinical status and cognitive functioning. The battery included a general measure of cognitive functions (Montreal Cognitive Assessment, MoCA), the geriatric depression scale (GDS), one test of “fluid” intelligence (digit symbol; Wechsler 1997), and one test of “crystallized” intelligence (similitude subtest; Wechsler 1997).

### Intervention

Two tasks were used for training: a visual detection task and an alphanumeric equation task. Both tasks were run on Compaq Pentium d530 computers, and responses were given on the keyboard. In the visual detection task, 3 × 30 square-inch (7.6 × 76 cm^2^) red or white rectangles appeared randomly at the bottom of the computer screen for 500 ms each, interspaced by 250-ms intervals (ISI). Participants were asked to press the spacebar key every time the rectangle was red and were to do so as quickly and accurately as possible. For the alphanumeric equation task, stimuli consisted of equations (addition or subtraction) containing letters (from N to Z) and numbers (1 or 2) in the format *x* + (or −) *n = z*. Participants were asked to indicate whether the equation was true or false. The letter *x* corresponded to the starting point in the alphabet, the + or − sign indicated the direction of the equation, and *n* was the number of letters that separated the starting point from *z*. The equation was visually presented in the middle of the screen for a maximum period of 3,750 ms with 1,500-ms interstimulus intervals. The participants were asked to judge the veracity of the equation by pressing one of two keys: the “F” key with the left index finger when the equation was false, and the “J” key with the right index finger when the equation was true. For example, the equation N + 2 = P is true because P is two letters after N in the alphabet, whereas the equation S − 1 = Q is false because one letter down from S in the alphabet is R and not Q. False equations were created by presenting a response that was one letter away (plus or minus) from the correct response. In the two divided attention training conditions (FIXED and VARIABLE), each block contained 20 equations, half of which were false and half of which were correct. Each equation appeared with five rectangles, including one to three red rectangles. Thus, 40 % of the rectangles were red, with a total of 20 to 100 rectangles per block, depending on the participant’s speed. The trial length was defined as the time required for participants to solve the equation. As soon as the participants responded to the equation, the next equation appeared and the trial was terminated. Thus, visual targets were only presented during the time participants took to solve the equation. This ensured that the participants were in a state of divided attention during the entire period. If a participant did not complete the alphanumeric equation within the required period of time, the next equation was presented immediately and the trial was considered as failed. Accuracy (AC) and reaction time (RT) were recorded for both tasks. Each training session comprised 13 blocks of 20 trials of the task. The more specific content of each block depended on the training condition as described below.

In the *variable divided attention training condition* (VARIABLE), participants were asked to perform both tasks simultaneously and to vary their allocation priorities across the series of blocks. Prior to each block, instructions informed the participants as to how much attention should be given to each task. There were three different levels of attentional allocation priority: 80 % Equation, 50 % Equation; and 20 % Equation. The 80 % Equation instruction condition indicated that the participants should allocate 80 % of their attention to the alphanumeric equation task and 20 % to the visual detection task. For the 50 % Equation instruction condition, the participants had to allocate an equal amount of attention to both tasks. Finally, for the 20 % Equation instruction condition, 20 % of the participants’ attention was asked to be on the alphanumeric equation task and 80 % on the visual detection task. The instructions were visually presented on the screen and read aloud to the participants. To enable better understanding, instructions were supported by an illustration of a rectangle-shaped box divided into two colored parts of different proportions, representing the percentage of attention required by each task. After each block, a histogram was presented to the participants indicating their baseline level for the training session (as measured earlier by the focused attention condition) and the targeted AC threshold according to the emphasis instructions. For example, if a participant responded correctly on 75 % of the alphanumeric equations in the focused attention condition, their AC threshold to attain in the 80 % Equation emphasis instruction would be 60 %. Before displaying their actual performance on the histogram on the computer screen, the participants were asked to draw their own estimate on the paper histogram. In this manner, the participants were informed as to whether they had attained the requested priority proportion to allow them to better adjust the emphasis at the next block. Each session comprised nine blocks in which the participants had to combine both tasks. To provide a baseline, the participants completed two blocks of each task in the focused attention condition at the beginning and end of each session.

In the *fixed divided attention training condition* (FIXED), participants were asked to complete the two tasks simultaneously and to give the same amount of attention to both tasks. Thus, they were asked to allocate 50 % of their attentional resources to the visual detection task and 50 % to the alphanumeric equation task. Each session comprised nine blocks where the participants had to combine both tasks. To provide a baseline, the participants completed two blocks of each task in the focused attention condition at the beginning and end of each session.

Finally, in the *Single task training condition* (SINGLE), participants performed both tasks individually with focused attention. To equate the number of blocks with the other two training conditions, it was composed of six blocks of one task and seven blocks of the other task. The number of blocks for each task alternated between sessions, so that the participants would receive the same amount of exposure to both tasks over the course of the whole training program. The starting task at session 1 was counterbalanced across the participants.

### Outcome measure

#### Primary outcome measure

Participants were asked to perform the visual detection and alphanumeric equation tasks separately (focused attention) and in combination (divided attention). The material was similar to that used in training, except that the equations contained letters from a different part of the alphabet (A to M rather than N to Z) to reduce potential practice effects due to familiarization with the letter position in the alphabet. Each condition (focused and divided) was presented in four blocks of 24 trials (for a total of 96) following an ABA design. Participants first completed each task with focused attention, followed by three blocks of the dual-task condition (80 % Equation, 50 % Equation, 20 % Equation). The two tasks were then completed again with focused attention. No feedback was given during the task.

#### Generalization measures

Generalization of training effects was measured with the N-back task, with a 1-back and a 2-back condition. For the N-back task, a series of letters were presented visually in the center of the screen. Letters appeared sequentially for 500 ms, with an interstimulus interval of 2,500 ms. In the 1-back condition, participants were asked to judge whether the letter was the same as that presented just one position before for the 1-back condition or two positions before for the 2-back condition. Each condition was presented in four blocks of 45 trials, 15 of which were targets. The order of presentation of the blocks followed an ABBA design. For the 2-back condition, the number of isolated trials (i.e., ABHBD) and embedded trials (i.e., ABHBH) was equivalent in each block to equate the level of difficulty. AC and RT for the correct answers were tested separately for each condition (1-back and 2-back).

### Design

Participants were randomized to one of the three training conditions, stratified by education and age to equate the three groups on those variables. Randomization was performed by an independent research technician. Training was provided in six 1-h sessions over 2 weeks. The outcome measures were assessed 1 week prior to the first training session and 1 week following the last training session. Two versions were available for the N-back task, and therefore, different versions were used in the pre- and post-sessions with order counterbalanced across the participants.

## Results

### Demographic and clinical data

Five participants were excluded for technical difficulties with the recording of their responses. The characteristics of the 37 remaining participants are presented in Table 1. Participants allocated to the three training groups were first compared on their sociodemographic and clinical characteristics using ANOVAs, with group (SINGLE, FIXED, and VARIABLE) as a between-subject factor. The three training groups were comparable for age, *F*(2, 34) = 0.12, *p* = .88; educational level, *F*(2, 34) = 0.58, *p* = .56; and performance on clinical measures.

**Table 1:** Mean scores for age, education, and clinical measures

	SINGLE (*n* = 12)	FIXED (*n* = 13)	VARIABLE (*n* = 12)	*F*	*p* value
Age	68.67 (8.28)	69.85 (5.96)	68.83 (5.24)	0.12	0.89
Education	14.75 (3.39)	15.15 (2.58)	16.17 (3.90)	0.58	0.56
MoCA	27.83 (1.64)	27.31 (1.60)	27.33 (2.57)	0.27	0.76
GDS (/15)	1.58 (1.44)	1.31 (1.25)	2.58 (3.48)	1.08	0.35
Similarities (WAIS-III)	12.58 (1.44)	12.23 (1.88)	12.58 (1.73)	0.18	0.84
Digit symbol	12.83 (2.55)	11.77 (1.53)	11.58 (2.02)	1.29	0.29

Standard deviations are in parentheses

### Dependent variables

Accuracy (AC) and reaction time (RT) were used as dependent variables in the focused attention condition of the visual detection and alphanumeric equation task. RTs less than 150 ms and greater than 4,000 ms were excluded, as well as RTs for commission errors. Because there were many dependent variables, a divided attention cost was computed by combining the RT and AC for each task in the divided attention condition relative to the focused attention condition, with the following equation: {[(RT divided − RT single) / RT single] + [(AC single − AC divided) / AC single]}. In the equation, RT single and AC single represent performance in the focused attention condition for RT and AC. RT divided and AC divided represent performance in the divided attention conditions (80 % Equation, 50 % Equation or 20 % Equation) for RT and AC. This divided attention cost represents the proportional loss of performance in the divided attention condition as a function of performance in focused attention. Thus, the formula controls for baseline performance and provides a measure of divided attention performance. When participants had a longer RT or lower AC in focused versus divided attention, this was scored as zero to avoid a negative attentional cost score.

### Pre-training

**Fig. 1:**
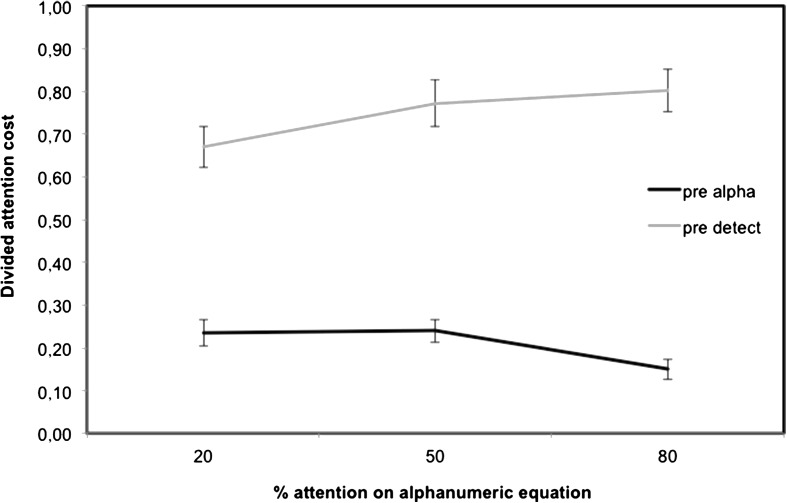
Divided attention cost for each task as a function of emphasis instruction (20 % Equation, 50 % Equation, and 80 % Equation) in pre-intervention (*error bars* represent standard error)

To assess whether there were group differences prior to training in spite of the randomization, divided attention cost during the pre-training session was first analyzed. The divided attention cost scores for each task as a function of emphasis instructions are displayed in Fig. 1. We performed a mixed ANOVA using divided attention cost as a dependent variable, emphasis (80 % Equation, 50 % Equation, or 20 % Equation) and task (alphanumeric equation; visual detection) as within-subject factors, and group (SINGLE, FIXED, and VARIABLE) as a between-subject factor.

The ANOVA showed no main effect of group (*p* = .62) and no interaction involving group, indicating that the three groups had similar baseline performance prior to training (*p* = .34, .86, and .58, respectively). A main effect of task was found *F*(1, 34) = 16.39, *p* < .001, as participants had an overall higher dual-task cost in the visual detection task (*M* = 0.75) compared to the alphanumeric equation task (*M* = 0.21). This effect was qualified by a task × emphasis interaction, *F*(2, 34) = 11.92, *p* < .001. Decomposition of the interaction indicated a significant emphasis effect for both the visual detection (*p* < .001) and the alphanumeric equation tasks (*p* < .001), but it can be seen that it goes in the opposite direction as would be expected. Follow-up tests revealed that the participants had a higher dual-task cost on the alphanumeric equation task in both the 20 % Equation (*M* = 0.23) and the 50 % equation emphasis instructions (*M* = 0.24) conditions than in the 80 % Equation (*M* = 0.15) instructions condition (*p* = .04 and .009, respectively). For the visual detection task, participants had a lower dual-task cost in the 20 % Equation (*M* = 0.67) compared to both 50 % Equation (*M* = 0.77) and 80 % Equation (*M* = 0.79) instruction conditions (*p* = .04 and .009, respectively). Thus, as shown in Fig. 1, prioritizing a task—whether it is the alphanumeric equation or the visual detection task—results in a decrease of dual-task cost on the task relative to a condition in which the two tasks are instructed to be equally emphasized.

To assess whether the three groups differed in focused attention prior to training, separate ANOVAs were computed for each task on the AC and RT recorded at pre-training, using group (SINGLE, FIXED, and VARIABLE) as a between-subject factor. Table 2 shows the pre-training performance of each training group on the two tasks in the focused attention condition. The ANOVA for the alphanumeric equation task shows that the three training groups had similar performance prior to training for both AC and RT, (*p* = .85 and .39, respectively). Similar results were found for the analysis of the visual detection task, which revealed no main group effect for either AC or RT (*p* = .55 and .09, respectively).

**Table 2:** Accuracy (AC) (%) and reaction time (RT) (ms) for alphanumeric equation task and visual detection task in the focused attention condition in pre-training and post-training

	Pre		Post	
	AC	RT	AC	RT
Alphanumeric equation				
SINGLE	84.1 (3.1)	2,383 (121.0)	86.8 (3.1)*	2,307 (127.0)*
FIXED	75.2 (4.6)	2,315 (105.0)	90.0 (3.6)*	2,146 (84.0)*
VARIABLE	77.3 (5.5)	2,466 (73.0)	89.8 (2.0)*	2,154 (100.0)*
Visual detection				
SINGLE	82.3 (8.3)	493 (19.0)	86.8 (7.7)	539 (37.0)
FIXED	92.6 (6.0)	438 (13.0)	99.3 (0.4)	449 (21.0)
VARIABLE	93.8 (5.2)	494 (30.0)	98.8 (0.7)	468 (13.0)

Standard deviations are in parentheses

*p < .01, main group effect

### Training effects

#### Focused attention

Table 2 shows the pre- and post-training performance of each training group on the two tasks in the focused attention condition. To assess the effects of training on task performance, separate mixed ANOVAs were computed for each task on AC and RT, using time (pre- and post-training) as a within-subject factor and group (SINGLE, FIXED, and VARIABLE) as a between-subject factor. The ANOVA showed a main effect of time on RT for the alphanumeric equation task *F*(1, 34) = 9.83, *p* < .001, indicating that the task was completed more rapidly following training compared to before training. There was neither a main group effect nor a time × group interaction, indicating that after having received training, all three groups had faster RT on the alphanumeric equation task completed under focused attention (*p* = .29 and .39, respectively). Similarly, when analyzing AC for the alphanumeric equation task, we found a main effect of time *F*(1, 34) = 14.8, *p* < .001 and no main effect of group or time × group interaction (*p* = .96 and .56, respectively). The analysis of RT for the visual detection task revealed no main time or group effects and no time × group interaction (*p* = .54, .55, and .21, respectively). Similarly, analysis of AC for the visual detection task revealed no main time or group effects and no time × group interaction (*p* = .10, .24, and .91, respectively). Thus, the three groups showed no gains from pre- to post-training in AC and RT on the visual detection task in the focused attention condition.

#### Divided attention and attentional control

**Fig. 2:**
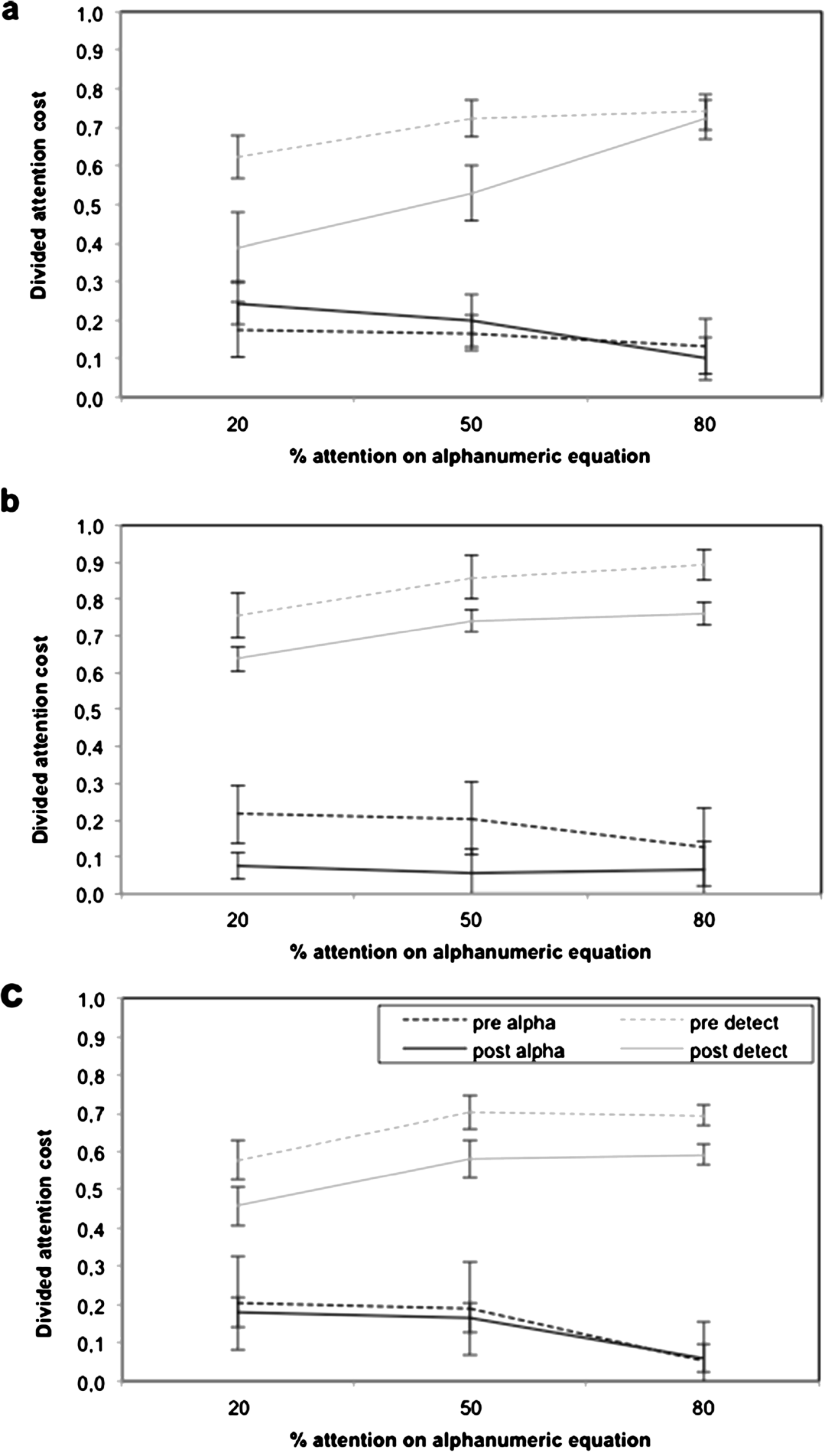
Divided attention cost for each task as a function of emphasis instruction (20 % Equation, 50 % Equation, and 80 % Equation) in divided attention for the VARIABLE (**a**), FIXED (**b**), and SINGLE (**c**) training group in pre- and post-intervention (*error bars* represent standard error)

The divided attention cost scores for each task as a function of emphasis instructions are displayed in Fig. 2. Divided attention cost scores were analyzed with a mixed ANOVA using time (pre- and post-training), emphasis (80 % Equation, 50 % Equation, or 20 % Equation), and task (alphanumeric equation, visual detection) as within-subject factors and group (SINGLE, FIXED, and VARIABLE) as a between-subject factor. The time × emphasis × task × group interaction was significant, *F*(1, 34) = 3.26, *p* < .001. To identify the source of the interaction, ANOVAs were computed separately for each group with the variables time, emphasis, and task.

For the VARIABLE training group, a significant time × emphasis × task interaction, *F*(2, 33) = 5.17, *p* < .001, was found. This was due to the presence of an emphasis × task interaction in post-training, *F*(2, 33) = 18.23, *p* < .001, but not in pre-training (*p* = .26). Examination of Fig. 2a and mean comparisons revealed that for both tasks, performance did not vary as a function of the priority emphasis instruction before training. However after training, the dual-task cost varies as a function of emphasis for both the alphanumeric equation and the visual detection tasks, but in opposite direction. After training, the main effect of emphasis found for the alphanumeric equation task, *F*(2, 33) = 8.83, *p* < .001, revealed that the dual-cost in the 80 % Equation (*M* = 0.10) emphasis instruction condition was smaller than in the 50 % Equation (*M* = 0.20) and 20 % Equation emphasis instruction condition (*M* = 0.24) (*p* = .03 and .001, respectively). The main effect of emphasis on visual detection at post-training, *F*(2, 33) = 14.13, *p* < .001, revealed a smaller dual-cost in the 20 % Equation (*M* = 0.39) than in the 50 % Equation (*M* = 0.53) and 80 % Equation (*M* = 0.39) (*p* = .03 and .002, respectively). Thus, the participants were better able to prioritize the visual detection task (20 % Equation emphasis) after training, when this was required. As a result and as shown on Fig. 2a, there is a significant 37 % dual-cost reduction from pre- (.62) to post-training (.39) for the visual detection task in the condition requiring emphasis on the detection task (20 % Equation; *p* = .02). There was also a significant 28 % dual-cost reduction in the 50 % Equation emphasis instruction condition from pre- (.73) to post-training (.53) (*p* = .001). These results indicate that from pre- to post-training, the participants improved their ability to vary the level of attention placed on each task in response to the instructions.

For the FIXED training group, a main time effect, *F*(1, 34) = 6.97, *p* < .001, was found, indicating that the participants improved their divided attention cost from pre- to post-training. Indeed, as shown in Fig. 2b, the participants lowered their dual-task cost from pre- to post-training, regardless of both tasks and instructions. There was also a significant emphasis × task interaction *F*(2, 33) = 5.17, *p* < .001. Decomposition of the interaction indicated that the emphasis’ main effect was significant only for the visual detection task (*p* < .001), due to a smaller dual-task cost in the 20 % Equation (*M* = 0.70) compared to both the 50 % Equation (*M* = 0.80) and 80 % Equation (*M* = 0.83) instructions (*p* = .02 and .05, respectively). Importantly, there was no time × emphasis × task interaction, indicating that the participants did not improve their ability to either divide or vary their level of attention after training (*p* = .35).

For the SINGLE training group (Fig. 2c), the emphasis × task interaction was significant, *F*(2, 33) = 6.02, *p* < .001. Decomposition of the interaction indicated that the emphasis effect was significant for both alphanumeric equation, *F*(2, 33) = 7.30, *p* < .001, and visual detection, *F*(2, 33) = 4.55, *p* < .001, but that the patterns differed. In the alphanumeric equation task, the participants had a lower dual-task cost in the 80 % Equation than the other two conditions (*p* = .02 and .02, respectively). In the visual detection task, the participants had a lower dual-task cost in the 20 % Equation than in the other two conditions (*p* = .02 and .03, respectively). Importantly, there was no main effect of time or time × emphasis × task interaction, indicating that the participants did not improve their ability to either divide or vary their level of attention after training (*p* = .81).

### Far-transfer measure

We performed separate mixed ANOVAs for AC and RT and the 1-back and 2-back conditions, using time (pre- and post-training) as a dependent variable and group (SINGLE, FIXED, and VARIABLE) as a between-subject factor. Results are presented in Table 3. There was no main effect of time or time × group interaction on the 1-back or 2-back conditions when using AC as a variable. Analysis on RT for the 1-back condition revealed a time × group interaction, *F*(2, 32) = 3.99, *p* = .003. Participants in the VARIABLE and FIXED training group significantly improved their completion time (*p* = .005 and *p* = .002, respectively), whereas no improvement was found for the SINGLE training group. Analysis on for the 2-back condition revealed a main effect of time, *F*(1, 32) = 39.59, *p* < .001, with all group performing more quickly after training. Group differences, however, were found in pre-training for AC and RT on the 1-back and 2-back conditions. Analysis on RT for the 1-back condition revealed a main group effect, *F*(2,34) = 8.16, *p* < .001. Post-hoc comparisons revealed that the VARIABLE training group was slower in pre-intervention compared to both FIXED and SINGLE groups prior to training (*p* = .04 and .03, respectively). For the 2-back condition, the analysis showed a main group effect, *F*(2,34) = 8.16, *p* = .003. Post-hoc comparisons revealed that the VARIABLE group was slower than the FIXED group in pre-intervention (*p* = .02). No group differences were found on AC for both 1-back and 2-back conditions (*p* = .81 and .44, respectively).

**Table 3:** Accuracy (%) and reaction time (ms) for the 1-back and 2-back conditions in pre-training and post-training

	1-back		2-back	
	Pre	Post	Pre	Post
Reaction time				
SINGLE	734.85 (131.95)	716.29 (94.14)	907.04 (232.60)	801.73 (199.68)**
FIXED	739.69 (127.54)	658.58 (126.87)*	856.65 (115.54)	729.25 (153.97)**
VARIABLE	950.18 (156.33)	828.54 (184.09)*	1,027.71 (176.90)	898.18 (110.05)**
Accuracy				
SINGLE	88.19 (5.60)	93.11 (3.21)	80.51 (10.73)	85.31 (10.56)
FIXED	87.97 (4.18)	94.94 (2.21)	85.92 (6.84)	91.46 (5.34)
VARIABLE	91.47 (6.56)	86.51 (7.85)	89.11 (7.85)	83.01 (9.44)

Standard deviations are in parentheses

*p < .05, main time effect; **p < .01, main group effect

**Fig. 3:**
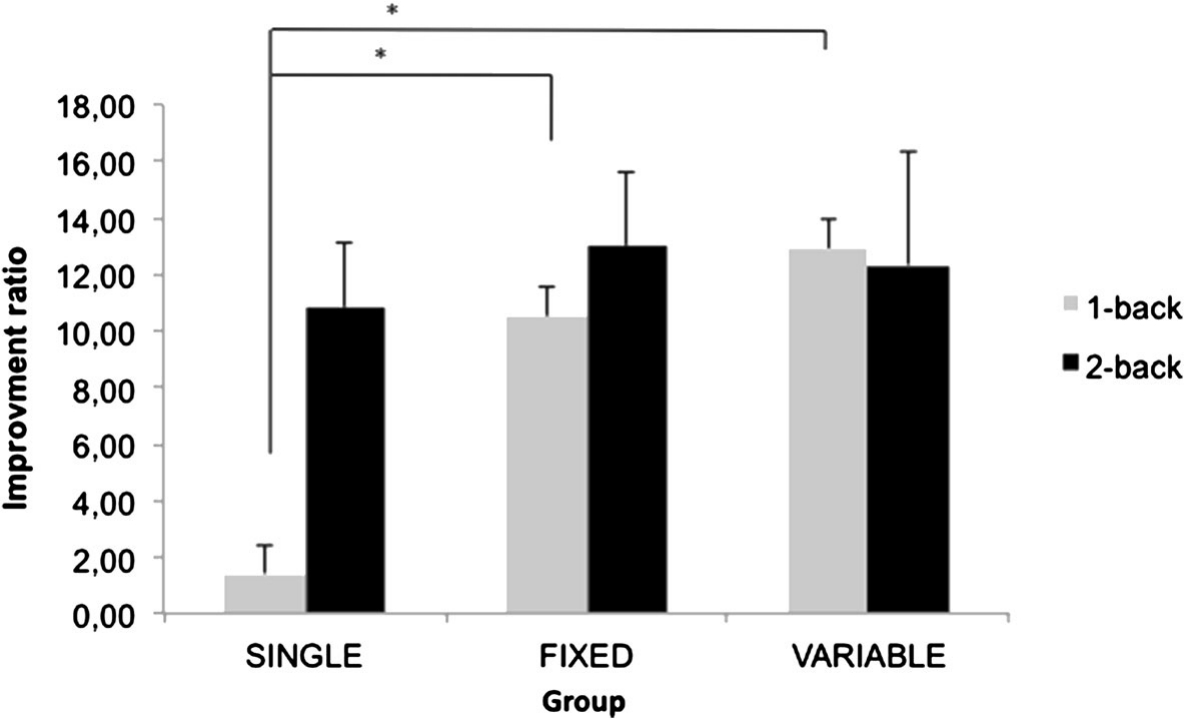
Reaction time improvement ratio [(Post − Pre) / Pre) × 100]) for the 1-back and 2-back conditions for SINGLE, FIXED, and VARIABLE training groups expressed in absolute value (*error bars* represent standard error). *p <* .05

Considering the group differences in pre-intervention, an improvement ratio was computed on RT and AC for both conditions of the generalization measure (1-back and 2-back) with the following equation: [(Post − Pre) / Pre) × 100]. This decrement indicates the improvement from pre- to post-training, controlling for the individual’s performance in pre-training. An improvement ratio on AC and RT for the 1-back and 2-back conditions is presented in Fig. 3. Separate ANOVAs were computed on the AC and RT improvement ratio for both 1-back and 2-back conditions, using training group (SINGLE, FIXED, and VARIABLE) as a between-subject factor. The analysis on AC showed no significant effects of group for the 1-back or the 2-back conditions (*p* = .50 and .19, respectively). When analyzing RT, a main group effect was found for the improvement ratio of the 1-back condition, *F*(2, 34) = 3.89, *p* = .031. The improvement ratio was larger in the VARIABLE (*M* = 12.93) and FIXED (*M* = 10.53) training group relative to the SINGLE training group (*M* = 1.42) (*p* = .02 and .03, respectively). There was no effect of group for the 2-back condition (*p* = .47*)*.

To assess whether a ceiling effect in the SINGLE training group could account for the results on RT, we computed correlations between RT at pre-training and the magnitude of the training effect and found a significant negative correlation (*r* = −0.51, *p* < .05). Thus, faster participants during training showed lower training effects, which supports the possibility that a ceiling effect is what may have prevented us from observing a training effect on the 1-back test in that group.

## Discussion

There were two goals in this study: to compare and identify the most efficient attentional training formats that produce the largest benefit for older adults and to assess whether efficacy transfers to distal measures. Older adults were randomized to three types of attentional training conditions: (1) *variable training* (VARIABLE), where participants practiced two tasks concurrently and varied their allocation priorities across a series of blocks; (2) *fixed attention training* (FIXED), in which participants practiced the two tasks concurrently and allocated the same amount of attention to both task; and (3) *single task training* (SINGLE), where participants practiced each task individually with full attention. Participants were assessed before and after training in focused and divided attention, using two tasks similar to the ones administered during training. Indeed, one of the goals and strengths of this study was to assess whether older adults were better able to modify their attentional priority in accordance to external demands (task instructions in the present case). Assessing the effect of training on modulation requires an analysis of all instruction conditions. We used dual-task cost as an outcome, as it takes into account the performance level in focused attention. Thus, the presence of a time effect, without an interaction with emphasis, was thought to reflect the training effect on divided attention abilities. In turn, improvement of controlled attention abilities (i.e., changing attentional priorities in response to specific environmental demands) was expected to result in a differential dual-task cost as a function of condition and, thus, as a time × emphasis × task interaction. The participants also completed a working memory task (N-back task; 1-back and 2-back conditions) to measure whether improvements would transfer to a task implicating the cognitive mechanisms expected to be improved with training (i.e., attentional control).

Results indicate that the different attentional training formats improve different aspects of attention that are highly coherent with the cognitive processes presumed to be enhanced by each training. It was hypothesized that the VARIABLE training condition increases the ability to control attention. This is confirmed by the finding of an improved ability to modify allocation priority as a function of task instructions. Furthermore, the extent to which the participants comply with task instructions in pre- and post-training is clearly documented. Indeed, even though the participants were prioritizing the alphanumeric equation task over the visual detection task in all three emphasis instructions in pre-training, the participants still tried to comply with the instructions by slightly lowering their dual-task cost on the task that needed to be prioritized. More interestingly, this effect was highlighted only in the VARIABLE training group after training. As a result, the participants considerably lowered their dual-task cost on the visual detection task after training. They showed the opposite effect when the instructions required that the alphanumeric equation be emphasized. Thus, participants in the VARIABLE group enhanced their dual-task coordination and management skills after training. This improvement reflects an increased ability to switch attentional priorities and increased metacognition abilities.

The outcome is strikingly different in the FIXED condition, in which participants were only asked to practice dual-tasking. In that case, the participants showed an overall attentional cost reduction after training but were not better able to vary their attentional emphasis across the two tasks. Finally, practice on individual tasks (SINGLE) resulted in better performance in the focused attention condition on the alphanumeric equation task; however, participants who received this training did not improve their divided attention and were not better able to control their attention.

Of note, however, is that most previous studies have used the 50-50 dual-task emphasis condition as their critical outcome variable. When using this condition as an outcome, we found that the FIXED and VARIABLE training improved performance but not the SINGLE training. This is consistent with the finding reported by previous investigators (Bherer et al. 2005, 2008) and further extent their finding by showing that it is not found in a control SINGLE training condition.

The results found with VARIABLE and FIXED training are coherent with what is reported in a number of previous studies (Gagnon and Belleville 2012; Kramer et al. 1995; Lee et al. 2012; Voss et al. 2012), which indicate that VARIABLE training produces greater improvements on executive coordination skills than FIXED training programs. Two studies, however, have reported no difference between VARIABLE and FIXED training conditions (Bherer et al. 2005, 2008). One obvious explanation is that those studies have used the 50-50 priority condition as their main outcome and as we showed, both training conditions improve this variable. A number of other procedural variations could also explain the divergent findings across studies. One difference is the type of task the participants were asked to combine. Indeed, Bherer et al. (2005, 2008) used simple auditory and visual discrimination tasks that were presented discretely and at fixed temporal intervals. In the present study, the participants performed a combination of self-paced and force-paced tasks as well as a task involving complex processing (alphanumeric equation). It is possible that varying priorities is most beneficial in settings in which there is more freedom to coordinate the two tasks. Another difference is relative task complexity or salience. In the present study, the participants reported that the alphanumeric equation task was more salient than the detection task prior to training. Indeed, dual-task cost was lower in the alphanumeric equation than in visual detection task, indicating that the participants favored the former over the latter. As aforementioned, the dual-task condition used a combination of tasks that differed in their level of complexity and attentional demand—one being a more complex task drawing more resources than the other—in order to simulate real-life situations, as tasks executed in divided attention are rarely equivalent. This posed particular challenges to the participants when they were asked to switch their attentional priority and emphasize detection (20 % Equation emphasis instruction). Interestingly, however, this condition was particularly sensitive to VARIABLE training, as it showed larger changes. Thus, differences in salience might modulate differences in attentional control or modulation capacities. Also, training attentional control may be particularly well designed for dual-tasking in conditions of differential salience. For example, it might be more efficient to improve attentional control involved in driving while engaging in complex conversation, rather than that involved in driving while listening to the news on the radio.

One important component of this study was the inclusion of a control training condition in which participants practiced both tasks individually with the same intensity and assessing whether this contributed to improved performance when combining them. This was motivated by models of executive control (Shallice 1994), which suggest that combining automatized processes is easier than combining demanding ones (i.e., novel information). Thus, one reasonable prediction is that becoming more proficient in the task through practice would increase one’s ability to combine them. It is critical to better understand the source of improvement in divided attention following dual-task training. Indeed, when practicing dual-tasking, participants gain practice in the individual tasks, which could make them easier to combine. It is thus important to make sure that the dual-tasking is bettered over and above the improved ability on the individual tasks. Results indicate that this is the case: participants in the SINGLE training condition improved their RT and AC in the alphanumeric equation task but did not improve their ability to divide their attention between the alphanumeric and visual detection tasks. Thus, improvement in dual-tasking does not result merely from participants developing an expertise with individual tasks.

The results found for the FIXED and SINGLE training groups are in line with a recent study that used a driving video game (Anguera et al. 2013). Young and older adults were asked to drive a car while simultaneously detecting a visual signal. Participants were randomly assigned to one of three training groups: a multitasking training (MTT), in which they were asked to perform both tasks concurrently in divided attention; a single task training (STT), where participants performed both tasks individually; and a no-contact control (NCC) group. After training, participants in the MTT training group showed a reduced multitasking cost from pre- to post-training, compared to the STT and NCC groups, with gains persisting up to 6 months. This reinforces the idea that a FIXED training format (or MTT) enables participants to perform better on both tasks concurrently. Our results under the SINGLE training condition is consistent with findings by Anguera et al. (2013) showing that enhanced multitasking ability was not solely the result of enhanced component skills, obtained by both the STT and MTT training groups, but rather a function of learning to resolve interference generated by the two tasks when performed concurrently. This suggests that it is possible to train specific dual-task coordination processes and that they are independent of those involved when practicing both tasks individually. The results are therefore consistent with the notion that the type of training, rather than solely the amount of practice, may be the best facilitator of skilled performance (Ericsson 2007; Ericsson et al. 1993). As was the case of the study of Anguera et al., the present findings offer behavioral evidence that targeted cognitive training programs could potentially benefit healthy older adults and enhance specific cognitive abilities.

Another important issue is whether the benefits of training generalize to other stimuli and tasks. Indeed, we questioned whether transfer would be greater for VARIABLE training over FIXED and SINGLE training. We hypothesized that VARIABLE training would transfer more to the 2-back than to the 1-back condition because it was more demanding at the executive level. In fact, attentional control or the ability to coordinate and monitor information processing is viewed as highly implicated in the executive component of working memory (McCabe and Hartman 2008; Miyake et al. 2000).

We measured training transfer on the N-back task (1-back and 2-back condition), which involves online monitoring, updating, and the manipulation of information within working memory (Owen et al. 2005). We found a complex set of intervention effects. On the 1-back condition, the VARIABLE and FIXED training formats resulted in larger improvements than the SINGLE format, which did not result in significant improvement from pre- to post-training. On the 2-back condition, all training groups improved their performance after training, including the SINGLE condition (see Fig. 3). This goes against our hypothesis of larger gains in the 2-back condition. One possibility that could account for this result is the ceiling effect observed in the SINGLE training group, as participants were extremely fast prior to training. The three training groups might have improved in both the 1-back and 2-back conditions had there not been a ceiling effect. If so, the improvement in all groups may be due to the fact that they all practiced on alphanumeric equation, a task involving working memory abilities. It is also possible that these observed gains are solely due to test-retest, as participants completed the tasks twice (prior to and after training), and there was no no-contact group to assess this possibility.

In summary, our results indicate post-training changes on working memory following attentional training. These results are in line with other studies reporting transfer from similar training programs to distal generalization measures (Bherer et al. 2005, 2008; Gagnon and Belleville 2012). Although a large number of studies report that transfer effects in dual-task training appear limited to near modality transfer or dual-task contexts, the present study demonstrates the possibility of relatively far transfer effects of training on broader working memory abilities. However, it is important to highlight that the result pattern differed from our predictions, and future studies will be required to determine whether the effects reflect the actual transfer or whether they are due to test-retest improvement. Furthermore, additional research is needed to further assess the breadth of those transfer effects, in particular whether the strategies participants learned during training or their improved capacities generalize to their everyday life activities. There are tools, such as self-administered questionnaires (Zanardo et al. 2006) and real-world safety tasks like driving simulators (Gaspar et al. 2013), that allow us to measure the impact of interventions on the complex activities of daily living. Virtual reality is also gaining in popularity, as it enables researchers and clinicians to create situations that simulate the complexities of daily life, while also allowing relatively solid experimental control. We are presently including this in our training procedure, as it may be one of the best strategies for enabling participants to transfer their attentional control abilities to a dual-task environment that is more representative of real-life settings.

In the current study, we found that it is possible to obtain selective effects, depending on the type of training used and that these effects may generalize differently to untrained cognitive abilities. Our results can have far-reaching implications considering the increasing amount of effort put toward developing training programs that target older adults. A number of commercialized products [Brain Fitness Program (Posit Science); Brain train, Cogmed (Pearson); Cognifit (Cognifit personal coach)] aim to prevent or reverse the effects of aging on cognition (for a review see Jak et al. 2013) by training a variety of cognitive abilities such as attention, memory, processing speed, inhibition, and multitasking. Our findings indicate that selection of the training approach is not neutral and can determine the magnitude of effects obtained. Current commercialized programs could benefit from a more fine-tuned approach to multitasking training.

It is important to address some limitations in this study. First, the number of participants per training group was small. Although our sample size proved to be sufficient to find a robust training effect, it might have been possible to detect more subtle differences with larger groups, particularly regarding our transfer task. Second, our study did not include booster sessions or long-term follow-ups to assess durability of the training effect. It will be critical to examine whether these improvements are maintained or fade over time. An additional limit concerns the validity of the generalization measures in terms of ecological value; the tasks selected did not represent actual activities of daily living.

In summary, our findings confirm that attentional control capacities of older adults are highly plastic and can be improved when appropriate training is provided. However, not all training programs have the same effect. Our results are in line with other studies, showing benefits of the VARIABLE training over the FIXED training in enhancing executive coordination skills. Furthermore, our findings demonstrate the importance of individual practice when the tasks used involve complex processes. Finally, this study underlines the fact that the type of training is critical in determining the impact on the target cognitive ability and the degree of generalization to untrained tasks.
